# Searching for the one(s): Using Probiotics as Anthelmintic Treatments

**DOI:** 10.3389/fphar.2021.714198

**Published:** 2021-08-09

**Authors:** Maria Priscila Saracino, Cecilia Celeste Vila, Pablo César Baldi, Daniel Horacio González Maglio

**Affiliations:** ^1^ Cátedra de Inmunología, Facultad de Farmacia y Bioquímica, Universidad de Buenos Aires, Buenos Aires, Argentina; ^2^ Instituto de Estudios de la Inmunidad Humoral (IDEHU), CONICET-Universidad de Buenos Aires, Buenos Aires, Argentina

**Keywords:** probiotics, helminths, inflammation, type 2 immune response, regulatory immune response

## Abstract

Helminths are a major health concern as over one billion people are infected worldwide and, despite the multiple efforts made, there is still no effective human vaccine against them. The most important drugs used nowadays to control helminth infections belong to the benzimidazoles, imidazothiazoles (levamisole) and macrocyclic lactones (avermectins and milbemycins) families. However, in the last 20 years, many publications have revealed increasing anthelmintic resistance in livestock which is both an economical and a potential health problem, even though very few have reported similar findings in human populations. To deal with this worrying limitation of anthelmintic drugs, alternative treatments based on plant extracts or probiotics have been developed. Probiotics are defined by the Food and Agriculture Organization as live microorganisms, which, when consumed in adequate amounts, confer a health benefit to the host. It has been proven that probiotic microbes have the ability to exert an immunomodulatory effect both at the mucosa and the systemic level. The immune response against gastrointestinal helminths is characterized as a type 2 response, with high IgE levels, increased numbers and/or activity of Th2 cells, type 2 innate lymphoid cells, eosinophils, basophils, mast cells, and alternatively activated macrophages. The oral administration of probiotics may contribute to controlling gastrointestinal helminth infections since it has been demonstrated that these microorganisms stimulate dendritic cells to elicit a type 2 or regulatory immune response, among other effects on the host immune system. Here we review the current knowledge about the use of probiotic bacteria as anthelmintic therapy or as a complement to traditional anthelmintic treatments. Considering all research papers reviewed, we may conclude that the effect generated by probiotics on helminth infection depends not only on the parasite species, their stage and localization but also on the administration scheme.

## Introduction

Helminths have co-evolved with mammals and infect over one billion people worldwide, mostly in non-industrialized countries (Hotez et al., 2008). Generally, helminths have complex life cycles, involving different stages and hosts. Most species of parasitic helminths occupy more than a single niche in a human host during their life cycle and, in most cases, helminths produce chronic infections. Despite worldwide efforts, the development of vaccines providing long-term protection against helminths has been hampered by multiple life cycle stages, antigenic variation between them, evasion mechanisms and immunomodulation strategies (Bobardt et al., 2020; Drurey et al., 2020). At present, only a few vaccines against helminths are commercially available with a high level of protection (>90%) and they are applied only to ruminants (Claerebout and Geldhof 2020). Therefore, a major approach to control helminth infections in livestock is periodic chemotherapy with anthelmintic drugs. However, uncomplete drug treatment schemes, high rates of post-treatments reinfections and the rise of anthelmintic resistance makes a dangerous combo for the increase of helminth infections among livestock with the consequent impact on human health (Hotez et al., 2008).

Probiotics are defined as live microorganisms that, when administered in adequate amounts, confer a health benefit on the host (Hill et al., 2014). These benefits include the prevention of health problems, such as diarrhea, irritable bowel syndrome, inflammatory bowel disease and allergic disorders such as atopic dermatitis (Islam 2016). The mechanisms of action of probiotics involve colonization and normalization of perturbed intestinal microbial communities, competitive exclusion of pathogens and bacteriocin production, modulation of enzymatic activities related to metabolization of a number of carcinogens and other toxic substances, and production of volatile fatty acids, which play a role in the maintenance of energy homeostasis and regulation of functionality in peripheral tissues (Bermudez-Brito et al., 2012; Plaza-Diaz et al., 2019). Probiotics also reinforce intestinal barrier function and mucin production and modulate the activity of gut-associated lymphoid tissue and the immune system (Plaza-Diaz et al., 2019). Likewise, probiotics may interfere with the physiology of parasites in the gut. Furthermore, their secreted products may have anthelmintic effects and can reduce the virulence of many parasites and for this reason probiotics may be an integral part of helminth parasite control strategies (Berrilli et al., 2012). Here we review the current knowledge about the use of probiotic bacteria as anthelmintic therapy or as a complement to traditional anthelmintic treatments.

## Immune Response Against Helminths

In general terms, the immune response in helminth infections is a type 2 immune response which is characterized by the production of interleukin (IL)-4, IL-5, IL-9, IL-10, and IL-13 (Allen and Maizels 2011). Adaptive immune cells including CD4^+^ T helper 2 (Th2) cells and B cells innate immune cells such as basophils, eosinophils, mast cells and innate lymphoid cells (ILCs) are important sources of type 2 cytokines and are important effector cells (Gause et al., 2020).

The epithelial cell barrier not only represents the first line of defense against helminths but also provides signals to initiate type 2 immune response. As helminths invade epithelial barriers and migrate through tissues, they cause considerable damage. The cell death of host cells is associated with the release of damage-associated molecular patterns (DAMPs), which trigger signaling pathways that contribute to the initiation of the type 2 response. Tissue damage is sensed by mucosal epithelial cells which promote the secretion of alarmins like IL-25, thymic stromal lymphopoietin (TSLP), and IL-33 (Wiedemann and Voehringer 2020). These alarmins induce activation and differentiation of type 2 immune cells which then release several other cytokines like IL-4, IL-5, IL-9, and IL-13. Epithelial cells also express a set of cytokines that educate dendritic cells (DCs) in promoting adaptive Th2 cell immunity and activate ILC2, basophils, eosinophils and mast cells; epithelial cells can also express chemokines such as CCL17, CCL22 and eotaxins (CCL11, CCL24 and CCL26) recruiting DCs, eosinophils, basophils, mast cells, and CD4^+^ T cells (Hammad and Lambrecht 2015). ILC2 are important in this type of response since they are inducer and effector cells and, like CD4^+^ T lymphocytes, express the transcription factor GATA3 and secrete IL-5, IL-9, and IL-13 at the beginning of the infection (Klose and Artis 2016; Kouchkovsky et al., 2017; Gurram and Zhu 2019).

IL-4 and IL-13 induce the proliferation of goblet cells, which secrete mucus and resistin-like molecule-beta (RELMβ). IL-4 induces IgE production; then, IgE-antigen immune complexes bind to high-affinity IgE receptors (FcεRI) on basophils and mast cells, causing the degranulation and release of several proinflammatory mediators, such as histamine, heparin and serotonin (Tantisira et al., 2007; Kubo 2018). IL-5 is responsible for the activation and recruitment of eosinophils, and IL-9 causes mast cell activation. Altogether, immune cells and the secreted cytokines coordinate parasite expulsion by increasing fluid and mucus production, encapsulation and barrier formation, epithelial cell turnover, smooth muscle cell contraction, and production of anthelmintic effector molecules, such as RELM-β (Babu and Nutman 2019). The type 2 cytokines mentioned also stimulate repair of tissues damaged by parasites, which control inflammatory processes and promote tissue remodeling and restructuring (Faz-López et al., 2016; Shapouri-Moghaddam et al., 2018).

Helminths have been shown to modulate/regulate the host response to their own benefit (parasite-specific immunoregulation) by releasing immunomodulatory molecules that evoke a regulatory phenotype among innate and adaptive immune cells (McSorley and Maizels 2012; Navarro et al., 2016; Wu et al., 2017; Gazzinelli-Guimaraes et al., 2018; Ryan et al., 2020). For this reason, and in the hygiene hypothesis context, helminth-derived products have been tested for treating autoimmune diseases (Yazdanbakhsh et al., 2002; R. M.; Maizels et al., 2014; Stiemsma et al., 2015). However, chronic helminth infections may induce allergic diseases as they enhance type 2 inflammation (Herz et al., 2000; Hurst et al., 2001; Demirci et al., 2003; Maizels and Yazdanbakhsh 2003).

Although helminth parasites are universal in inducing all or most of these type 2 effector pathways, in the host the specific effector pathway mediating protection varies between different parasites, lifecycle stages, and site of infection.

## Mechanism of Action of Probiotics

To discuss the role of probiotics in parasite infections it is necessary to describe their general effects on the gut environment and their immunomodulatory capacity.

As mentioned, probiotics are live microorganisms that, when administered in adequate amounts, confer a health benefit on the host. Among these probiotics, one of the more important groups is that of lactic acid bacteria. These microorganisms have the ability to produce large quantities of lactic acid, which inhibits the growth of pathogenic bacteria, and have been used to produce dairy foods for centuries (Metchnikoff 1908; Mackowiak 2013; Fisberg and Machado 2015). The former genus *Lactobacillus*, subdivided into 25 genera (including 23 new ones), includes different species with proven probiotic activity, such as *Lacticaseibacillus casei*, *Lacticaseibacillus rhamnosus*, and *Lactobacillus delbrueckii* (Zheng et al., 2020). Moreover, other genera like *Bifidobacterium*, *Enterococcus* and *Streptococcus,* also include strains with probiotic capacity.

The ability to promote health benefits by these bacteria resides in direct effects on other microorganisms in the gut lumen as well as in the modulation of immune and non-immune cells (Plaza-Diaz et al., 2019). Regarding the direct effects, besides the production of lactic acid, these bacteria can proliferate in the gut, preventing the colonization by different pathogens. Moreover, probiotics produce and secrete different bacteriocins, peptides that affect the growth of bacteria, fungi, parasites, and viruses (Hernández-González et al., 2021). They also produce short-chain fatty acids (SCFA), like butyrate and acetate, and branched-chain fatty acids (BCFA), such as isobutyrate and 2-methylbutyrate. These volatile fatty acids help the host cells, including immune ones, to maintain energy homeostasis.

Regarding the effects produced specifically on the immune system, they are triggered by interactions between the microorganisms and cellular receptors. The latter are present both in epithelial and immune cells, being the dendritic cells (DC) that emit cytoplasmic processes into the gut, an essential mediator of probiotics’ effects (Sánchez et al., 2017). The bacterial surface molecules recognizable through specific receptors include the peptidoglycan, lipoteichoic and teichoic acids (surface molecule of gram-positive bacteria), surface proteins, and different glycan residues present in surface molecules. These molecular targets are recognized by receptors such as toll-like receptor 2 (TLR2), TLR6, nucleotide-binding oligomerization domain-containing protein 1 (NOD1), NOD2, and others. The receptor-target interaction mediates effector responses that usually depend on the gut environment. Under physiological conditions, these responses tend to promote immune tolerance, a lack of specific effector inflammatory responses, and the avoidance of damage to the microorganisms and the surrounding tissue. However, activated DC can also prime T cells, inducing effector Th1, Th2, or Th17 responses against different targets, such as microorganisms and cancer cells (both local and distant ones) (de Moreno de LeBlanc et al., 2007). Probiotics can also modify immune responses through the secretion of different molecules. In this way, the role of lactic acid production on gut immunity has been described. *In vitro* assays showed that lactate decreases inflammatory responses of DC and macrophages, an effect that correlates with reduced intestinal inflammation in a murine colitis model (Iraporda et al., 2015; Iraporda et al., 2016). Besides lactic acid, SCFA also affect immune cell metabolism and the overall immune response (Tan et al., 2014). However, probiotics can also modulate immune cells in an indirect way. When enterocytes recognize molecular targets on probiotics, these cells are capable of secreting a wide variety of pro- and anti-inflammatory cytokines which ultimately affect immune cells (Corthésy et al., 2007). The effects mentioned do not require live probiotics to be produced. It has also been observed that heat-killed bacteria as well as molecules isolated from probiotics, such as lipoteichoic acid, can exert some of the effects observed for live microorganisms (Friedrich et al., 2017).

The local effects produced by probiotics on the gut environment and the gut-associated lymphoid tissues (GALT) have allowed their use as a treatment or as a supplementary therapy on different gastrointestinal diseases. Such uses go from treating gut dysbiosis (produced by antibiotic therapy, diarrhea, food intake, or others) to restoring the microbial balance in the gut (McFarland 2014) or modulating the immune response in inflammatory diseases and cancer. Briefly, probiotics’ use in inflammatory bowel diseases, being ulcerative colitis (Dang et al., 2020) and Crohn’s disease (Lichtenstein et al., 2016) the most frequent pathologies, has shown different grades of efficacy (Orel and Trop 2014). Whereas there was some efficacy in regulating inflammation in ulcerative colitis, there was no evidence of significant beneficial effect in Crohn’s disease. However, the immune system modulation induced by probiotics is beneficial in the case of gastrointestinal tumors. In this case, probiotics exert a prophylactic effect (observed both in animal models and in epidemiological studies) and protect the gut against side effects of antitumor treatments (Drago 2019). Some of the modulatory effects are related to the cytokines induced in the probiotic-treated animals, including TNF-α, IFN-γ, and IL-10 (de LeBlanc and Perdigón 2004).

Nevertheless, the immune modulation induced by oral probiotics is not limited to the gastrointestinal tract. These effects can impact on distant organs, such as the lungs and the skin (Friedrich et al., 2017). This can be observed in atopic diseases, such as asthma and atopic dermatitis (AD). Regarding the respiratory atopic disease, an interesting meta-analysis showed that probiotics’ administration reduced the number of episodes in treated children, with a concomitant reduction in IL-4 production and an increase in IFN-γ levels, showing a bias of the T helper response towards a Th1 profile. However, no statistical differences were found in other outcomes of the pathology (Lin et al., 2018). According to these findings, a meta-analysis done by Wei and colleagues showed that the use of probiotic supplementation did not associate with a lower risk of asthma in infants (Wei et al., 2020). In the case of AD, a significant number of clinical trials revealed AD prevention with probiotics’ consumption in children (Meneghin et al., 2012). Moreover, the authors also reported the therapeutic effectiveness of the treatment with different beneficial microorganisms once the pathology was installed (Meneghin et al., 2012). In a more recent meta-analysis, Rusu and colleagues argue that despite these results there is not enough data regarding optimal dosing, optimal time to start treatment and duration necessary to show beneficial effects (Rusu et al., 2019). Finally, the immunomodulatory capacity of probiotics was also demonstrated in skin cancer studies. *Lactobacillus* spp. lipoteichoic acid produced a reduction in squamous cell carcinomas in chronically UV-exposed mice, reinforcing Th1 response (Weill et al., 2013). Moreover, this treatment was also effective in preventing UV-induced immunosuppression (Friedrich et al., 2019). In addition, the oral treatment with *Bifidobacterium* spp. was as effective as PDL-1 blocking monoclonal antibody in affecting melanoma’s growth in a mouse model (Sivan et al., 2015).

Overall, probiotics affect the balance of gut microbiota and modify the availability of molecular targets for immune receptors and soluble metabolites, leading to modulation of innate and adaptive immune responses. These modulations are induced both at the local and the systemic levels (Sivan et al., 2015).

## Probiotics as Complement/Treatment Against Helminth Infections

Experimental evidence on the use of probiotics to treat parasite infections is limited. It is important to highlight the lack of blind, placebo controlled, clinical trials. Consequently, most of the published work commented hereafter was developed in different animal models, including experimental models (typically mouse) and susceptible animals (such as pigs).

Evidence about the role of gastrointestinal bacteria on parasite infection can be found in the correlation between microbiota composition and parasite infection. Reynolds et al. reviewed the effects of the presence of parasites on the microbiota, and vice versa (Reynolds et al., 2015). Interestingly, the absence of microbiota (in germ-free mice) hampers parasite infection. Moreover, the presence or administration of some *Lactobacillus* strains promote helminths colonization or persistence. The authors highlight that *Lactobacillus* spp. tend to decrease type 2 responses and increase Tregs, as mentioned, and propose that those mechanisms may explain the results observed in germ-free mice.

Here, we present experimental and epidemiological evidence about the role of probiotics and commensal bacteria on different helminth infections.

### Nematodes

#### *Ascaris* spp.

Hosts contract *Ascaris* spp. infection *via* the faecal-oral route; when embryonated eggs are ingested, larvae penetrate the gastrointestinal tract and enter the blood stream. Through the blood stream the larvae are carried to the liver and heart, then enter pulmonary circulation and are released in the alveolar space, where they grow and molt. From the respiratory system the larvae are coughed up, swallowed, to finally reach the small intestine, where they mature to adult male or female worms. After mating, female worms release eggs that contaminate faeces.

Different works were published studying the effect of probiotics during this helminth infection, including some in pigs infected with *Ascaris suum* (Jang et al., 2017; Solano-Aguilar et al., 2018). The first report, by Jang et al., described the use of *L. rhamnosus* GG (LGG) as a probiotic treatment of the infection. Moreover, the authors included flavonol-rich cocoa powder (CP) as a supplement, due to the immunomodulatory capacity of the flavonols such as catechin and epicatechin present in the extract. The treatment was applied during 5 weeks prior to parasite challenge, and the animals were evaluated 17 days after inoculation. No significant differences in intestinal parasite content (L4 larvae) were observed, but a delay in intestinal expulsion of parasitic larvae from the intestine was registered in the CP + LGG group. No changes in serum specific IgG2 were obtained with the probiotic treatment. Interestingly, the LGG treatment alone induced modifications of cytokine and chemokine transcription (decrease of IL-1β, IL-13, and CCL26) in the tracheobronchial lymph nodes but not in the mesenteric lymph nodes (Jang et al., 2017).

Using the same model of host and parasite, Solano-Aguilar et al. evaluated the effect of *Bifidobacterium animalis* subspecies *lactis* with the modulation of the intestinal immune response. The probiotic treatment began on the sows and continued in newborn piglets for 2.5 months, when they were inoculated with *A. suum*. Even though they did not observe changes in the number of intestinal L4 larvae at 17 days post infection (p.i.), an increase in specific antibodies was detected (serum IgA and ileum fluid IgG1 and IgG2). Moreover, jejunal mucosa from infected pigs showed a characteristic decrease in glucose absorption and an increase in the secretory response to histamine, both being attenuated by the probiotic treatment. Finally, the eosinophilia promoted by the parasite was decreased by *B. animalis* subspecies *lactis*, without affecting the expulsion of the worms (Solano-Aguilar et al., 2018).

In another work, the therapeutic ability of inactivated *Bacillus thuringiensis* overexpressing Cry5B protein (paraprobiotic with anthelmintic properties) was tested in *A. suum* infection models. The paraprobiotic intoxicated *A. suum* larvae *in vitro* and was highly effective against intestinal *A. suum* infections in a mouse model. In pigs, a single oral dose of this paraprobiotic reduced the parasite burden by 96% during *A. suum* infections (Urban et al., 2021). This strategy results in an attractive alternative for the control other helminth infections.

All these data suggest that during *Ascaris* spp. infection depending on the treatment realized with probiotics, the effects on parasitological and immunological parameters could be beneficial, negative, or neutral. The scarcity of studies on the effects of probiotics against this helminth infection clearly indicates the need of further research on this topic.

#### *Trichuris* spp.

*Trichuris trichiura* is one of the most common gastrointestinal nematodes. In infected children, trichuriasis is associated with malnutrition, growth stunting, and reduced educational performance, whereas in adults, it is related to anemia, reduced worker productivity, and/or low-birth-weight babies (Bethony et al., 2006). The cycle initiates by ingestion of contaminated water or food with embryonated eggs, which hatch in the intestine to release infective L1 larvae. L1 migrate to the caecum and colon and undergo four molts to adult worms. Adult females produce eggs, which are excreted in the feces. Infective eggs in the environment are subsequently ingested. *Trichuris muris* in the mouse has provided a useful and relevant model system with which to explore immunity to *T. trichiura* in man due to their homology at the genomic and transcriptomic level (Klementowicz et al., 2012; Foth et al., 2014). Infection of mice with *T. muris* drives polarized T helper cell (Th) responses, which associate with resistance (Th2) or susceptibility (Th1) (Klementowicz et al., 2012). B cells are important in the development and maintenance of the protective immune response to *T. muris* (Sahputra et al., 2019).

The role of probiotics inducing regulatory immune responses was discussed above. However, implications of this mechanism against parasite infection are not necessarily straightforward. McClemens et al. showed the effects of *L. rhamnosus* (JB-1) administration against *Trichuris* spp. infection, highlighting the role of IL-10 on those effects. Susceptible and resistant mouse strains (AKR and C57BL/6, respectively) were inoculated with *T. muris* and fed with live and γ-irradiated probiotics. The procedure was conducted also in IL-10 KO mice. The number of worms in the caecum was reduced by live *L. rhamnosus* at 10, 14, and 21 days post-inoculation (*vs*. control), but the effect was lost when dead bacteria were used. The increased parasite expulsion induced by this probiotic was also lost in IL-10 KO mice, showing the important role of this regulatory cytokine. Moreover, the AKR mouse strain, susceptible to develop chronic infections with *T. muris*, also showed a decrease in parasite burden after *L. rhamnosus* treatment. An increase in IL-10 production was observed in these mice, reinforcing the results about the role of this cytokine in the expulsion of the nematode (McClemens et al., 2013).

In another work, Dea-Ayuela et al. studied the effect of an oral treatment with either viable or dead *L. casei* (ATCC 7469) before *T. muris* infection. The results in the treated groups were not encouraging; both conditions favored the infection as the mean number of L3 larvae recorded were significantly higher than in challenged untreated controls. Regarding the immune response, viable *L. casei* reduced the levels of fecal IgA induced by challenge infection, decreased the cellular response (diminished proliferation of MLN cells with mitogens), and reduced IFN-γ, TNF-α, IL-4 and IL-13 in both MLN and PP compared to infected untreated mice (Dea-Ayuela et al., 2008).

When we compare both studies, results seem to depend on the species of probiotic used. While *L. rhamnosus* treatment had a protective effect on *T. muris* infection, critically dependent on IL-10, *L. casei* treatment diminished the immune response at both the cellular and humoral level, leading to a higher parasite load.

#### Trichinella spiralis

The life cycle of *T. spiralis* is completed in one host and there is no free-living stage. Transmission occurs when infected skeletal muscle containing muscle larvae (ML or L1) is consumed. In the stomach, ML are freed from muscle by digestion and move into the small intestine where they invade epithelial cells and migrate through the epithelial monolayer creating syncytia. ML molt four times becoming adult worms (AW) who mate in the epithelium. Female worms release newborn larvae (NBL) which enter the lymphatics and eventually reach the bloodstream. During their journey to their final ecotope, NBL can extravasate in any tissue, such as liver, lungs, or heart, but can only complete their development in skeletal muscle cells. When fully mature, each larva can infect a new host.

Clearance of AW from intestine is mediated by a potent type 2 response, which is characterized by increases in the numbers of lymphocytes, eosinophils, goblet cells and mast cells, and also by the switch to IgE and IgG1 isotypes (Ahmad and Bell 1991; Negrão-Corrêa 2001; Saracino et al., 2020). Regarding the NBL, it is known that they are killed by antibody-dependent cellular cytotoxicity (ADCC) both at systemic and tissue levels (Kazura and Grove 1978; Wang and Bell 1987; Venturiello et al., 1993; Gentilini et al., 2011; Falduto et al., 2014).

Bautista-Garfias et al. studied the ability of viable *L. casei* (ATCC 7469), administered by an intraperitoneal route, to induce resistance in mice against *T. spiralis* infection. The percent of reduction of adult worms in the intestine 5 days after *T. spiralis* infection observed in *L. casei*-treated animals, compared with those of the control group, fluctuated between 70.9 and 88.5%; and the reductions of larvae per gram of muscle tissue, evaluated at 30 days after infection, varied from 46.6 to 84.4% (Bautista-Garfias et al., 1999). Later, this group evaluated the effects of *L. casei* (ATCC 7469 live, heat-killed and culture supernatant) orally administered prior to oral challenge with *T. spiralis*. The treatment with live probiotic reduced AW numbers by 58%, whereas dead probiotic did it by 44% and culture supernatant by 32%. ML were also decreased by 70, 65 and 24% by live bacteria, dead bacteria, and culture supernatant, respectively (Bautista-Garfias et al., 2001). These results show that, in the response against *T. spiralis*, the immune mechanisms triggered by probiotics components are enough to induce the observed effects. Moreover, soluble mediators produced by *L. casei* are also effective, even though with a lower potency. Comparing administration routes, intraperitoneal versus oral, it seems that the parasitic load reductions were higher with administration by intraperitoneal route.

A more recent study of the group analyzed the effect of the intraperitoneal administration of *Lacticaseibacillus casei* Shirota strain in CD1 mice on the establishment of *T spiralis* AW, and on the generation of intestinal IgA anti-*T. spiralis* after challenge. From day 5 p.i., mice in *L. casei* Shirota group showed a significantly smaller number of AW and higher levels of IgA anti-*T. spiralis* than animals from the untreated group which suggest that *L. casei* Shirota would be protecting mice from *T. spiralis* infection (Martínez-Gómez et al., 2009)*.*

More recently, an interesting approach to evaluate the role of immune modulation and probiotics in *T. spiralis* infection was published. Wang et al. treated mice with both wild type and IL-4 recombinant *Lactiplantibacillus plantarum* NC8 prior to challenge with *T. spiralis*. A marked reduction of the infection-induced weight loss was observed with both treatments. However, wild type *L. plantarum* failed to reduce intestinal adult worms at day 7 post-infection, whereas the IL-4-expressing bacteria produced a significant decrease. Interestingly, the number of ML found at day 28 after parasite inoculation was significantly reduced by both treatments (being the effect of the recombinant bacteria more intense) (Wang et al., 2020). This work shows that *Lacticaseibacillus* spp. administration can partially reduce parasite burden, and that the effect is stronger in the presence of IL-4. However, these results contrast with those obtained by Temsahy et al. who found that serum IFN-γ concentration was raised in mice fed with *L. plantarum* P164 whether they were or not infected with *T. spiralis* (Temsahy et al., 2015). Actually, the authors attribute this IFN-γ increase to the bacterial peptidoglycan and not, as other authors, to the infection by *T. spiralis* (Dvorožňáková et al., 2011; Gentilini et al., 2011) or the combination of both stimuli. These authors also showed that the probiotic was able to colonize the gut after probiotic feeding, which they explained as a result of its adhesion ability.

Dvorožnáková et al. explored the effects of *Enterococcus faecium* CCM8558, *Enterococcus durans* ED26E/7, *Limosilactobacillus fermentum* CCM7421 and *L. plantarum* 17L/1 on cellular immunity. Mice treated with probiotic strains and then infected with *T. spiralis* showed a higher cellular response in terms of phagocytosis and respiratory burst (Dvorožňáková et al., 2016). Moreover, when the distribution of CD4^+^ and CD8^+^ cells was studied in the intestine they found that there was a higher number of CD8^+^ cells at the epithelia and increased numbers of CD4^+^ cells at lamina propria, which could contribute to the reduction in the number of adult worms in the host (Dvoroznakova et al., 2016). Regarding this point, Temsahy et al. found an increase in goblet cells hyperplasia in the intestines of mice treated with *L. plantarum* which could also explain the lower number of AW recovered from the gut as these cells are involved in AW expulsion (Temsahy et al., 2015).

Bucková et al. have shown that the parasite burden, the number of adult worms, the female fecundity and NBL were diminished when mice were treated with *E. faecium* CCM8558, *E. durans* ED26E/7 and *L. fermentum* CCM7421. Also, these probiotic strains reduced the female fecundity with the subsequent reduction in the number of NBL in the *in vitro* assays (Bucková et al., 2018).

#### *Toxocara* spp.

Humans are accidental hosts of *Toxocara* spp. who become infected by ingesting infective eggs or undercooked meat/viscera of infected hosts. After ingestion, the eggs hatch, and larvae that penetrate the intestinal wall are carried by the circulation to a variety of tissues (liver, heart, lungs, brain, muscle, eyes). While the larvae do not undergo any further development in these sites, they can cause local reactions and mechanical damage leading to clinical toxocariasis. *T. canis* causes larva migrans syndrome that induces an immune response characterized by blood eosinophilia, eosinophilic infiltration around larval sites of migration, specific antibody production (IgG and IgE) and a Th2 response (Ruiz-Manzano et al., 2019). Migrating larvae are attacked by host immune responses, resulting in local inflammation associated with eosinophilia and increased production of cytokines and specific antibodies. Although many *T. canis* infections are subclinical in nature, human toxocariasis can manifest itself as syndromes known as visceral larva migrans, ocular larva migrans, neurotoxocariasis, and covert or common toxocariasis (Taylor et al., 1988; Finsterer and Auer 2007; Pivetti-Pezzi 2009).

Regarding *Toxocara* spp. infection and probiotic treatment, several experimental reports are published. Most of them evaluated the parasite burden after pretreatment with probiotics in mouse models. Moreover, the direct effect of the microorganisms on parasite viability was evaluated *in vitro* in many of these works. *Saccharomyces boulardii* is a non-bacterial microorganism, closely related with *S. cerevisiae*, with probiotic properties which also is resistant to the adverse conditions of gastric and intestinal environments (Pais et al., 2020).

de Avila et al. (2012) showed that *S. boulardii* was able to reduce the number of larvae in Swiss mice infected with *T. canis* at both the acute and chronic phase of the infection. However, *in vitro* assays did not show a larvicidal effect on L3 larvae (de Avila et al., 2013) suggesting the necessity of a contact of *S. boulardii* with the intestinal mucosal or microbiota to mediate the observed *in vivo* effects. In a subsequent, the authors explored the effect of *S. boulardii* on the immune system in mice infected with *T. canis* (de Avila et al., 2016). The cytokine secretion in splenocytes from mice orally treated or not with *S. boulardii*, and later infected with *T. canis*, was evaluated. The study showed that diet supplementation with *S. boulardii* stimulates a Th1 response since IL-12 and IFN-γ genes transcription was elevated in both infected and not infected groups whereas IL-4 and IL-10 did not present any significant differences between treatments. In a later work, this group used a different infection model as mice were infected with larvae and not with *T. canis* eggs. This study not only did not reveal a significative reduction of *T. canis* larvae, but also showed that IL-12 transcription was below the threshold value in both supplemented and not supplemented infected mice. It is worth to notice that uninfected animals supplemented with the probiotic showed a significant increase in this cytokine transcription in the duodenum (de Moura et al., 2017). Taken together these results point out that *S. boulardii* anthelmintic properties will depend on the parasitic stage that is aimed to eliminate. Finally, these researchers studied the effect of *S. boulardii* when animals are infected by the ingestion of raw liver from chickens infected with *T. canis* larvae in order to emulate the natural infection. Mice that received diet supplemented with *S. boulardii* showed a reduction of 64.4% in the number of larvae recovered from the liver and 66.7% from the lungs as compared to those not treated with the probiotic.

The effects of *L. rhamnosus* (ATCC 7469) and *L. acidophilus* (ATCC 4356) on the experimental infection with *T. canis* were also investigated. The probiotic treatment was initiated before parasite challenge with *T. canis* embryonated eggs. These probiotics successfully reduced the number of migrating larvae found in liver at 48 h p.i. (52% reduction for *L. rhamnosus* and 58% for *L. acidophilus*). As previously found for *S. boulardii*, *L. rhamnosus* and *L. acidophilus* displayed their effects *in vivo* but did not exert a direct effect on the parasite *in vitro*, demonstrating once more that the interaction between the host and these bacteria is of paramount importance for the protective effects (de Avila et al., 2012; Walcher et al., 2018; Cadore et al., 2021).

Another probiotic evaluated regarding the prevention of *T. canis* infection was *E. faecalis* (CECT 7121). In this case, co-administration of probiotic and parasite led to a reduced number of larvae recovered from liver and lungs (Basualdo et al., 2007). However, if the parasite infection was done before probiotic treatment the effect was lost (Chiodo et al., 2010).

### Trematodes

#### *Schistosoma* spp.

During infection with *Schistosoma* spp. adult worms produce eggs which exit the host *via* urine or fecal matter. When schistosome eggs enter freely into the environment they hatch to produce miracidia that invade snails and develop along different stages. Cercariae travel from the intermediate snail host to the definitive mammalian host. Following attachment and skin penetration, cercariae transform to schistosomula which travel to the lungs, and then to the liver. Within the liver, worms develop into female and male adults that finally lodge within the portal and mesenteric vessels of the small intestine or the veins of the vesical and pelvic plexuses, from where the gravid females release eggs. Unfortunately, many bloodborne eggs become lodged within vascularized tissues and organs being the main cause of pathology following infection (Burke et al., 2009). The pathogenesis of schistosome infections and the morbidity associated with infection is due to a lethal combination of highly immunogenic eggs, a vigorous immune response and the various organs in which eggs become trapped.

To restrain the invading cercariae, innate and local stromal cells trigger an inflammatory cascade, with the release of macrophage inflammatory protein (MIP)-1, IL-6, IL-1, IL-12/23p40 and IL-18. Cercarial products can also directly stimulate production of cytokines, such as IL-4 and IL-10, which dampen the Th1 inflammatory response via their antagonistic effects on IL-12/23p40 production.

In the first weeks of murine *S. mansoni* infection, while the host is exposed to schistosomula and semi-mature schistosomes, a Th1-like immune response is observed. With the onset of egg deposition, however, a pronounced Th2 immune response comes up being characterized by high production of IL-4 and IL-13, IgE synthesis as well as eosinophilia and mastocytosis. Finally, when infection becomes chronic and egg production continues, a general down-modulation of immune reactivity develops, leading to a diminished Th2 response together with a smaller size of newly formed granulomas (Pearce and MacDonald 2002). The eggs induce a granulomatous host immune response largely characterized by lymphocytes (which mainly produce Th2 cytokines), eosinophils, and alternatively activated macrophages (Pearce and MacDonald 2002; Fairfax et al., 2012). This response eventually sequesters egg products, but it can also lead to severe hepatic fibrosis and portal hypertension.

In the case of schistosomiasis, the effectiveness of probiotic treatment was studied alone or combined with antiparasitic drugs. Zowail et al., 2012 evaluated the treatment of mice previously infected with *S. mansoni* with either *Bacillus coagulans*, praziquantel (drug of choice for the control of schistosomiasis) or a combination of both. They observed a 53% reduction in the number of adult worms using the probiotic alone, an 89% with the standard praziquantel treatment, and a 100% reduction with the combined treatment. Moreover, the number of eggs in the liver and intestine was also reduced in a similar way; for the liver, reductions of 47, 59 and 87% were observed, whereas for the intestine the reductions were 51, 53 and 71% (probiotic alone, praziquantel, and combined therapy, respectively). This work highlights the importance of evaluating the probiotics as a supplement for regular antiparasitic treatments (Mohamed et al., 2016). As other authors have demonstrated, infection by *S. mansoni* induces chromosomal aberrations and DNA damage (Shubber and Salih 1987) as well as praziquantel treatment (Montero and Ostrosky 1997) so in order to prevent this effect, El-Esawy et al. used *B. coagulans* as a complement of anti-parasitic treatment. When anthelmintic treatment was combined with *B. coagulans* a significant reduction in chromosomal aberrations induced by infection or praziquantel treatment was observed. This work highlights the importance of evaluating the probiotics as a supplement for regular antiparasitic treatments (El-Esawy 2012).

In a more recent work performed by El-Khadragy et al., the efficacy of a mixture of *L. acidophilus* (ATCC 4356) and *L. delbrueckii* subsp*.* bulgaricus (DSM 20080) was evaluated in a mouse model of *S. mansoni* infection. In this work, probiotics were administered either before or after parasite challenge. Moreover, the probiotic mix was applied in saline buffers and as a yogurt (allowing the probiotic to ferment milk for 5 h). Although results were also compared with praziquantel treatment, combined therapy was not evaluated. The authors observed a reduction in adult worm burden in mice treated with either probiotics or yogurt, both in administrations pre- and post-infection (68 and 60% for probiotics pre- and post-infection, and 72 and 64% for yogurt pre- and post-infection). Probiotics were almost as effective as praziquantel, which produced a 78% decrease in the number of adult worms. The reduction in liver eggs followed a similar pattern. Interestingly, *S. mansoni* infection increased the hepatic levels of MMP-9, lipid peroxidation, and NO, and decreased the levels of reduced glutathione. All these effects were prevented or reverted by probiotic treatment, as well as by praziquantel. Interestingly, the drug failed to decrease hepatic NO levels, whereas all probiotic treatments were successful (El-Khadragy et al., 2019).

De Fátima Macedo Santos et al. studied the effect of *Zymomonas mobilis* administration in mice divided into prophylactic and curative groups. Also, a mixed group was considered which received bacterial culture before and after *S. mansoni* infection. The total number of adult worms recovered was lower in the curative group, resulting in 61% protection. However, when prophylactic treatment was applied there was a non-significant reduction of adult worms. Surprisingly, animals belonging to the mixed group had an exacerbation of the infection, with a larger number of adult worms (De Fátima Macedo Santos et al., 2004).

Ghanem et al. used yoghurt containing *L. casei*, *L. acidophilus*, *L. plantarum* and *Limosilactobacillus reuteri* to fed mice before and after infection with *S. mansoni.* This strategy resulted in body weight gain as well as decreased spleen and liver weights to values close to controls. This probiotic yoghurt was found to display an immunomodulatory effect by stimulating an IgM response against soluble worm antigens as compared to the untreated control. While the infection increased AST, LDH and gGT activity in plasma, the addition of probiotic yoghurt led to a significant decrease of these enzymes in infected animals (Ghanem et al.,2005).

Taken together, these studies suggest that probiotics treatment may be used before or after infection with *Schistosoma* spp. However, as suggested by the study of De Fátima Macedo Santos et al., the combination of both treatments should be studied previously in each model to rule out potential adverse events such as increased parasitic load. This exacerbated infection may be due to an anergy state.

### Cestodes

#### *Echinococcus* spp.

The two species with clinical importance are *E. granulosus* and *E. multilocularis*. The life cycle of this cestode involves dogs and other canids as definitive hosts, and sheep and other herbivores as intermediate hosts. After ingestion by a suitable intermediate host eggs hatch in the small intestine and release an oncosphere that actively penetrates the intestinal wall and migrates through the circulatory and lymphatic system into several organs, particularly the liver and lungs. In these organs, the oncosphere develops into a metacestode (cyst) that gradually enlarges, producing protoscolices and daughter cysts that remain within the cyst. The definitive host is infected after ingesting the cyst-containing organs of the infected intermediate host. After ingestion, the protoscolices evaginate, attach to the intestinal mucosa and develop into adults.

Humans are accidental intermediate hosts and become infected via ingestion of eggs which hatch and develop into metacestodes (fluid-filled cysts, hydatids) in tissues, particularly the liver and lungs. Two essential mechanisms appear to be at the basis of the often long-lasting and asymptomatic co-habitation of the hydatid cyst and the intermediate host: immune evasion/modulation and protective immunity to re-infection. The latter is antibody- and complement-dependent (Dempster et al., 1992; Dempster et al.,1995; Heath and Lawrence 1996; Lightowlers 2010; Torben et al., 2012), and is enhanced in the presence of neutrophils (Rogan et al., 1992). A mixed Th1/Th2 response, together with high levels of IL-10, is evoked as shown by *ex vivo* stimulation of splenocytes with protoscoleces (PSC) extract. The production of IL-10 and IL-4 could be actively induced by the parasite to favor its establishment (Dematteis et al., 1999).

Regarding the effects of probiotics in echinococcosis, there are few but interesting studies. Yousif and Ali studied the effect of a mix of *L. acidophilus*, *L. casei* and *L. rhamnosus* in the immune response against infection with secondary hydatid disease as an antiparasitic immunomodulator in BALB/c mice. The bacteria were administered by intraperitoneal route in mice, before and after infections with *E. granulosus* protoscoleces. Many criteria were considered, including numbers, weight, diameter, and percentage reduction of hydatid cysts of treated mice as compared to infected animals not treated with probiotics. The study showed a decline in cysts, including their diameter, weight, and number in probiotic treated animals. The bacteria were applied at two different concentrations, both promoting the reduction in number, size and diameter of hydatid cysts 6 months post-infection (98.03% reduction). It may well be concluded that probiotic bacteria can be used as a therapeutic method against hydatidosis. Unfortunately, no immune analysis was done (Yousif and Ali 2020).

Vogt et al. presented a different approach: they developed and used a recombinant *Bacillus subtilis* strain, carrying two antigens from *E. granulosus*. This approach is based on the capacity of the recombinant bacteria to act as a delivery system for the vaccination antigens. The work was done in dogs and the response evaluated was the production of specific antibodies in the serum of the treated animals. Dogs generated a humoral response, mainly IgG, not only against E. granulosus peptides but also against some B. subtilis antigens (Vogt et al., 2018). However, it is interesting the use of a well-known and safe probiotic microorganism as delivery system, which may have an impact *per se* against a challenge with the parasite.

### Other Helminths

#### Hookworms

Hookworms are soil-transmitted nematode parasites that can reside for many years in the small intestine of their human hosts, where they suck blood and can cause iron deficiency anemia (Loukas et al., 2016). Two major species of hookworms infect humans: *Necator americanus* and *Ancylostoma duodenale*. During the life cycle of the hookworm eggs expelled in the feces of the infected host hatch in the environment, resulting in L1 larvae, which then molt twice to L3 (infective). L3 penetrate the skin of mammalian hosts. The larvae then enter the bloodstream, migrate through heart to the lungs, break through the alveoli, creep up the trachea and are swallowed, eventually residing in the small intestine to mature to adult worms. In the small intestine, adult hookworm mate and produce eggs that are passed in the feces, completing the life cycle.

Human hookworm infection generates a robust specific Th2 response, with some evidence of a systemic, but not mucosal hookworm-specific Th1 response (Gaze et al., 2012). Despite the predominance of Th2 cells and cytokines, and parasite-killing antibody isotypes (such as IgE), attempts to dislodge adult hookworms from the gut are mostly unsuccessful. Hookworms are potent inducers of regulatory immune responses that promote their survival and reproductive capacity. In humans, the expansion of regulatory T cells (Treg cells) has been reported (Wammes et al., 2014).

Regarding the use of probiotics to treat these infections, a study by Coêlho et al. analyzed the administration of *L. acidophilus* (ATCC 4536), *L. plantarum* (ATCC 8014), and *L. delbrueckii* (UFV H2B20) to control canine ancylostomiasis. The probiotic preparation was administered to naturally infected animals for 28 days, resulting in a reduction of eggs found in feces, as well as an increase in leukocyte and lymphocyte counts. It is important to mention that, before the treatment, red blood cells were below normal values in all dogs. The stabilization of the anemia in treated dogs, compared to its exacerbation in control animals, may be associated with the reduction of the number of eggs in the treated group (Coêlho et al., 2013).

#### H. polygyrus

*H. polygyrus* has a direct lifecycle with no intermediate hosts: eggs released in the feces of infected mice hatch in the environment producing L1 larvae, which molt twice to L3 larvae (infective stage), which are ingested by mice. L3 invade the intestinal mucosal layer molting into L4, which encyst in the muscle layer of the intestine and start maturing into adult parasites. Adult male and female worms mate in the lumen of the intestine, and gravid females produce eggs that are passed into feces.

*H. polygyrus* infection induces a strongly polarized Th2 response, which has been shown to be critical for the control and expulsion of the worm (Urban et al., 1991). A primary *H. polygyrus* infection induces IL-3, IL-4, IL-5, and IL-9 gene expression in the MLN and Peyer’s patches (Svetić et al., 1993). Mononuclear cells from MLN, spleen or lamina propria stimulated *in vitro* with parasite antigens released high amounts of IL-4, IL-5, IL-9, IL-10, and IL-13 (Finney et al., 2007; Setiawan et al., 2007; Rausch et al., 2008). At the cellular level, infection is accompanied by expanded regulatory T cell populations and B cell hyperstimulation. In most mouse strains, these act to block protective Th2 immunity (Rick M. Maizels et al., 2012).

Reynolds et al. found that administration of *Lactobacillus taiwanensis* (BL263) enhanced Treg frequencies which made animals more susceptible to *H. polygyrus* infection. Moreover, *H. polygyrus* raises *Lactobacillus* species abundance in the duodenum of C57BL/6 mice, which are susceptible to *H. polygyrus* infection, but not in BALB/c mice, which are relatively resistant. Sequencing of samples at the bacterial gyrB locus identified the principal *Lactobacillus* species as *L. taiwanensis* (Reynolds et al., 2014). This causal relationship between commensal bacterium and *H. polygyrus*, highlights the importance of the mutualistic relationship between a commensal microbe and a helminth parasite, which provides a different perspective on the interactions in the intestinal tract that we have seen in this work.

#### *Strongyloides* spp.

*Strongyloides* spp. are soil-transmitted helminths. The primary mode of infection is through contact with soil contaminated with free-living larvae. The latter penetrate the skin and migrate through the body, eventually finding the small intestine where they mature into adults and produce eggs. Unlike other soil-transmitted helminths, the eggs of these helminths hatch into larvae in the intestine. Most of these larvae will be excreted in the stool, but some of them may mature and re-infect the host either by burrowing into the intestinal wall, or by penetrating the skin around the anus.

Like other helminth infections, strongyloidiasis elicits a predominant Th2 immune response (Wilkes et al., 2007). During primary infection neutrophils and eosinophils are attracted by parasite components and kill the larvae through the release of granule products. B-cells produce both IgM and IgG that collaborate with neutrophils to kill worms (Bonne-Année et al., 2011).

In the case of *Strongyloides* spp., a work by Oliveira-Sequeira et al. evaluated the administration of viable *B. animalis* strain 04450B before the infection with *S. venezuelensis*. They found in probiotic-treated mice a decrease in the worm burden (33%) and egg output (21%) accompanied by a reduced intestinal damage (Oliveira-Sequeira et al., 2014). Unfortunately, there is no immune analysis done in this study.

#### Haplorchis taichui

The intestinal trematode *Haplorchis taichui* is a medically important parasite infecting humans and livestock. This parasite has an aquatic life cycle, using freshwater snails as the first and cyprinid fish as the second intermediate hosts, with definitive hosts being fish-eating mammals (Dzikowski et al., 2004; Nithikathkul and Wongsawad 2008).

In a different approach to study the relationship between parasite infections and gastrointestinal microorganisms, Prommi et al. analyzed stool samples from 1,047 volunteers from Thailand. A parasitological study was conducted, and 16s rRNA sequencing was performed to evaluate microbial diversity. While this is not a study about the role of probiotics in parasite infections, it contributes to highlight the relevance of microbiota balance regarding parasite infection. A high prevalence of the trematode *Haplorchis taichui* was found in the samples. Notably, the group of volunteers without parasite infections exhibited a higher bacterial diversity (α diversity) compared with the *H. taichui*-infected group. Moreover, differences in bacterial community composition were also found (β diversity). The authors concluded that *H. taichui* infection modifies microbiome (Prommi et al., 2020). However, it is possible to question us: does parasite infection succeed if microbiota is first affected by other reasons? An alternative explanation, however, could be that *H. taichui* is more likely to produce successful infections in individuals with an intestinal microbiota previously affected for other reasons.

## Conclusion

The use of probiotics as a treatment for helminth infection is an incipient research line with promising perspectives. Even though the papers discussed above show auspicious findings, they also raise additional questions. Regarding the host immune system, a detailed characterization of how the probiotic treatment affects the known immune mechanisms towards helminths is needed. As was mentioned before some features remain as open questions. For instance, which would be the best time to start treatment, right after the infection takes place or before? What is the optimal duration of the treatment? Once more, it would depend on the combination of parasite species and the probiotic strain (combination). Also, we have yet little information about which probiotics molecules, superficial or secreted, are responsible for the effects reviewed in this work. A fascinating aspect to explore would be the combined use of probiotics strains and anthelmintic drug treatments. Last but not least, it is important to highlight the lack of blind, placebo-controlled clinical trials as these treatments are expected to be applied to human or animal individuals.

We look forward to many more findings in this field as there is a wide range of possibilities to be explored that may deliver groundbreaking treatment strategies for helminth diseases.

## References

[B1] Ahmad A. Wang C. H. Bell R. G. (1991). A Role for IgE in Intestinal Immunity. Expression of Rapid Expulsion of *Trichinella spiralis* in Rats Transfused with IgE and Thoracic Duct Lymphocytes. J. Immunol. 146 (10), 3563–3570. 2026881

[B2] Allen J. E. Maizels R. M. (2011). Diversity and Dialogue in Immunity to Helminths. Nat. Rev. Immunol. 11 (6), 375–388. 10.1038/nri2992 21610741

[B3] Babu S. Nutman T. B. (2019). Immune Responses to Helminth Infection. Clin. Immunol., 437–447. 10.1016/b978-0-7020-6896-6.00031-4

[B4] Basualdo J. Sparo M. Chiodo P. Ciarmela M. Minvielle M. (2007). Oral Treatment with a Potential Probiotic (Enterococcus faecalisCECT 7121) Appears to Reduce the Parasite burden of Mice Infected withToxocara canis. Ann. Trop. Med. Parasitol. 101 (6), 559–562. 10.1179/136485907X193824 17716442

[B5] Bautista-Garfias C. R. Ixta O. Orduña M. Martínez F. Aguilar B. Cortés A. (1999). Enhancement of Resistance in Mice Treated with Lactobacillus Casei: Effect on Trichinella Spiralis Infection. Vet. Parasitol. 80 (3), 251–260. 10.1016/S0304-4017(98)00210-6 9950348

[B6] Bautista-Garfias C. R. Ixta-Rodríguez O. Martínez-Gómez F. López M. G. Aguilar-Figueroa B. R. (2001). Effect of Viable or deadLactobacillus Caseiorganisms Administered Orally to Mice on Resistance againstTrichinella Spiralisinfection. Parasite 8 (August), S226–S228. 10.1051/parasite/200108s2226 11484363

[B7] Bermudez-Brito M. Plaza-Díaz J. Muñoz-Quezada S. Gómez-Llorente C. Gil A. (2012). Probiotic Mechanisms of Action. Ann. Nutr. Metab. 61 (2), 160–174. 10.1159/000342079 23037511

[B8] Berrilli F. Di Cave D. Cavallero S. D'Amelio S. (2012). Interactions between Parasites and Microbial Communities in the Human Gut. Front. Cel. Inf. Microbio. 2 (November), 141. 10.3389/fcimb.2012.00141 PMC3499702 23162802

[B9] Bethony J. Brooker S. Albonico M. Geiger S. M. Loukas A. Diemert D. (2006). Soil-Transmitted Helminth Infections: Ascariasis, Trichuriasis, and Hookworm. The Lancet 367 (9521), 1521–1532. 10.1016/S0140-6736(06)68653-4 16679166

[B10] Bobardt S. D. Dillman A. R. Nair M. G. (2020). The Two Faces of Nematode Infection: Virulence and Immunomodulatory Molecules from Nematode Parasites of Mammals, Insects and Plants. Front. Microbiol. 11 (December), 1–13. 10.3389/fmicb.2020.577846 33343521 PMC7738434

[B11] Bucková B. Hurníková Z. Lauková A. Revajová V. Dvorožňáková E. (2018). The Anti-parasitic Effect of Probiotic Bacteria via Limiting the Fecundity of Trichinella Spiralis Female Adults. Helminthologia (Poland) 55 (2), 102–111. 10.2478/helm-2018-0010 PMC6799552 31662635

[B12] Burke M. L. Jones M. K. Gobert G. N. Li Y. S. Ellis M. K. McManus D. P. (2009). Immunopathogenesis of Human Schistosomiasis. Parasite Immunol. 31 (4), 163–176. 10.1111/j.1365-3024.2009.01098.x 19292768

[B13] Cadore P. S. Sousa N. F. G. C. d. Martins L. H. R. Hora V. P. d. Groll A. V. Moura M. Q. d. (2021). Protective Effect of the Probiotic Lactobacillus Acidophilus ATCC 4356 in BALB/c Mice Infected with Toxocara Canis. Rev. Inst. Med. Trop. S. Paulo 63, 1–6. 10.1590/s1678-9946202163009 PMC7845935 33533812

[B14] Chiodo P. G. Sparo M. D. Pezzani B. C. Minvielle M. C. Basualdo J. A. (2010). *In Vitro* and *In Vivo* Effects of Enterococcus Faecalis CECT7121 on Toxocara Canis. Mem. Inst. Oswaldo Cruz 105 (5), 615–620. 10.1590/S0074-02762010000500003 20835606

[B15] Claerebout E. Geldhof P. (2020). Helminth Vaccines in Ruminants. Vet. Clin. North America: Food Anim. Pract. 36 (1), 159–171. 10.1016/j.cvfa.2019.10.001 PMC7125739 32029181

[B16] Coêlho M. D. G. Coêlho F. A. d. S. Mancilha I. M. d. (2013). Probiotic Therapy: A Promising Strategy for the Control of Canine Hookworm. J. Parasitol. Res. 2013, 1–6. 10.1155/2013/430413 PMC3872410 24386558

[B17] Corthésy B. Gaskins H. R. Mercenier A. (2007). Cross-Talk between Probiotic Bacteria and the Host Immune System. J. Nutr. 137 (3). 64. 10.1093/jn/137.3.781s 17311975

[B18] Dang X. Xu M. Liu D. Zhou D. Yang W. (2020). Assessing the Efficacy and Safety of Fecal Microbiota Transplantation and Probiotic VSL#3 for Active Ulcerative Colitis: A Systematic Review and Meta-Analysis. PLoS ONE 15 (3), e0228846–16. 10.1371/journal.pone.0228846 32182248 PMC7077802

[B19] de Avila L. F. d. C. d. Conceição F. R. Telmo P. d. L. Dutra G. F. Santos D. G. d. l. Martins L. H. R. (2012). Saccharomyces Boulardii Reduces Infection Intensity of Mice with Toxocariasis. Vet. Parasitol. 187 (1–2), 337–340. 10.1016/j.vetpar.2012.01.002 22305116

[B20] de Avila L. F. D. C. de Leon P. M. M. de Moura M. Q. Berne M. E. A. Scaini C. J. Leivas Leite F. P. (2016). Modulation of IL-12 and IFNγ by Probiotic Supplementation Promotes Protection against Toxocara Canis Infection in Mice. Parasite Immunol. 38 (5), 326–330. 10.1111/pim.12314 26971490

[B21] de Avila L. F. d. C. d. Telmo P. d. L. Martins L. H. R. Glaeser T. A. Conceicao F. R. Leite F. P. L. (2013). PROTECTIVE EFFECT of the PROBIOTIC Saccharomyces Boulardii IN Toxocara canis INFECTION IS Not DUE to DIRECT ACTION on the LARVAE. Rev. Inst. Med. Trop. S. Paulo 55 (5), 363–365. 10.1590/S0036-46652013000500012 24037293 PMC4105076

[B22] de LeBlanc A. De. M. Perdigón G. (2004). Yogurt Feeding Inhibits Promotion and Progression of Experimental Colorectal Cancer. Med. Sci. Monitor 10 (4), 96–105. 15039638

[B23] de LeBlanc A. d. M. Matar C. Perdigón G. Perdigón G. (2007). The Application of Probiotics in Cancer. Br. J. Nutr. 98 (Suppl. 1), S105–S110. 10.1017/S0007114507839602 17922945

[B24] de Moura M. Q. da Silva Terto W. D. Jeske S. T. de Castro L. M. Pinto N. B. da Costa Avila L. F. (2017). Evaluation of the Transcription of Interleukin-12 in the Intestinal Mucosa of Mice Subjected to Experimental Toxocariasis and Supplemented with Saccharomyces Boulardii. Vet. Parasitol. 242 (May), 59–62. 10.1016/j.vetpar.2017.05.012 28606326

[B25] Dea-Ayuela M. A. Rama-Iñiguez S. Bolás-Fernandez F. Bolás-Fernandez F. (2008). Enhanced Susceptibility to Trichuris Muris Infection of B10Br Mice Treated with the Probiotic Lactobacillus Casei. Int. Immunopharmacology 8 (1), 28–35. 10.1016/j.intimp.2007.10.003 18068097

[B26] Dematteis S. Baz A. Rottenberg M. Fernández C. Orn2 A. Nieto A. (1999). Antibody and Th1/Th2-type Responses in BALB/c Mice Inoculated with Live or Dead Echinococcus Granulosus Protoscoleces. Parasite Immunol. 21 (1), 19–26. 10.1046/j.1365-3024.1999.00198.x 10081768

[B27] Demirci M. Yildirim M. Aridogan B. C. Baysal V. Korkmaz M. (2003). Tissue Parasites in Patients with Chronic Urticaria. J. Dermatol. 30 (11), 777–781. 10.1111/j.1346-8138.2003.tb00477.x 14684933

[B28] Dempster R. P. Harrison G. B. L. Berridge M. V. Heath D. D. (1992). Echinococcus Granulosus: Use of an Intermediate Host Mouse Model to Evaluate Sources of Protective Antigens and a Role for Antibody in the Immune Response. Int. J. Parasitol. 22 (4), 435–441. 10.1016/0020-7519(92)90144-A 1644518

[B29] Dempster R. P. Harrison G. B. L. Berridge M. V. (1995). Maternal Transfer of Protection from Echinococcus Granulosus Infection in Sheep. Res. Vet. Sci. 58 (3), 197–202. 10.1016/0034-5288(95)90101-9 7659840

[B30] Drago L. (2019). Probiotics and Colon Cancer. Microorganisms 7 (3), 66–11. 10.3390/microorganisms7030066 PMC6463067 30823471

[B31] Drurey C. Coakley G. Maizels R. M. (2020). Extracellular Vesicles: New Targets for Vaccines against Helminth Parasites. Int. J. Parasitol. 50 (9), 623–633. 10.1016/j.ijpara.2020.04.011 32659278 PMC8313431

[B32] Dvoroznakova E. Buckova B. Hurnikova Z. Revajova V. Laukova A. (2016). Distribution of CD4 and CD8 T Cells in the Small Intestine of Mice after Probiotic Treatment and Trichinella Spiralis Infection. Ann. Parasitol. 62 (Suppl. l), 8558.

[B33] Dvorožňáková E. Bucková B. Hurníková Z. Revajová V. Lauková A. (2016). Effect of Probiotic Bacteria on Phagocytosis and Respiratory Burst Activity of Blood Polymorphonuclear Leukocytes (PMNL) in Mice Infected with Trichinella Spiralis. Vet. Parasitol. 231, 69–76. 10.1016/j.vetpar.2016.07.004 27425573

[B34] Dvorožňáková E. Hurníková Z. Kołodziej-Sobocińska M. (2011). Development of Cellular Immune Response of Mice to Infection with Low Doses of Trichinella Spiralis, Trichinella Britovi and Trichinella Pseudospiralis Larvae. Parasitol. Res. 108 (1), 169–176. 10.1007/s00436-010-2049-x 20967464

[B35] Dzikowski R. Levy M. Poore M. Flowers J. Paperna I. (2004). Use of RDNA Polymorphism for Identification of Heterophyidae Infecting Freshwater Fishes. Dis. Aquat. Org. 59 (1), 35–41. 10.3354/dao059035 15212290

[B36] El-Khadragy M. F. Al-Olayan E. M. Elmallah M. I. Y. Alharbi H. M. Abdel Moneim A. E. (2019). Probiotics and Yogurt Modulate Oxidative Stress and Fibrosis in Livers of Schistosoma Mansoni-Infected Mice. BMC Complement. Altern. Med. 19 (1), 1–13. 10.1186/s12906-018-2406-3 30606163 PMC6318950

[B37] Fairfax K. Nascimento M. Huang S. C.-C. Everts B. Pearce E. J. (2012). Th2 Responses in Schistosomiasis. Semin. Immunopathol 34 (6), 863–871. 10.1007/s00281-012-0354-4 23139101

[B38] Falduto G. H. Vila C. C. Saracino M. P. Calcagno M. A. Venturiello S. M. Venturiello Stella. M. (2014). Trichinella Spiralis: Killing of Newborn Larvae by Lung Cells. Parasitol. Res. 114, 679–685. 10.1007/s00436-014-4233-x 25416332

[B39] Faz-López B. Morales-Montor J. Terrazas L. I. (2016). Role of Macrophages in the Repair Process during the Tissue Migrating and Resident Helminth Infections. Biomed. Res. Int. 2016, 1–11. 10.1155/2016/8634603 PMC5014929 27648452

[B40] Finney C. A. M. Taylor M. D. Wilson M. S. Maizels R. M. Maizels R. M. (2007). Expansion and Activation of CD4+CD25+ Regulatory T Cells in Heligmosomoides Polygyrus Infection. Eur. J. Immunol. 37 (7), 1874–1886. 10.1002/eji.200636751 17563918 PMC2699425

[B41] Finsterer J. Auer H. (2007). Neurotoxocarosis. Rev. Inst. Med. Trop. S. Paulo 49 (5), 279–287. 10.1590/S0036-46652007000500002 18026633

[B42] Fisberg M. Machado R. (2015). History of Yogurt and Current Patterns of Consumption. Nutr. Rev. 73 (Suppl. 1), 4–7. 10.1093/nutrit/nuv020 26175483

[B43] Foth B. J. Tsai I. J. Reid A. J. Bancroft A. J. Nichol S. Tracey A. (2014). Whipworm Genome and Dual-Species Transcriptome Analyses Provide Molecular Insights into an Intimate Host-Parasite Interaction. Nat. Genet. 46 (7), 693–700. 10.1038/ng.3010 24929830 PMC5012510

[B44] Friedrich A. D. Campo V. E. Cela E. M. Morelli A. E. Shufesky W. J. Tckacheva O. A. (2019). Oral Administration of Lipoteichoic Acid from Lactobacillus Rhamnosus GG Overcomes UVB‐induced Immunosuppression and Impairs Skin Tumor Growth in Mice. Eur. J. Immunol. 49 (11), 2095–2102. 10.1002/eji.201848024 31334839 PMC6854281

[B45] Friedrich A. Paz M. Leoni J. González Maglio D. (2017). Message in a Bottle: Dialog between Intestine and Skin Modulated by Probiotics. Ijms 18 (6), 1067. 10.3390/ijms18061067 PMC5485927 28598354

[B46] Gause W. C. Rothlin C. Loke P. n. (2020). Heterogeneity in the Initiation, Development and Function of Type 2 Immunity. Nat. Rev. Immunol. 20 (10), 603–614. 10.1038/s41577-020-0301-x 32367051 PMC9773851

[B47] Gaze S. McSorley H. J. Daveson J. Jones D. Bethony J. M. Oliveira L. M. (2012). Characterising the Mucosal and Systemic Immune Responses to Experimental Human Hookworm Infection. Plos Pathog. 8 (2), e1002520. 10.1371/journal.ppat.1002520 22346753 PMC3276555

[B48] Gazzinelli-Guimaraes P. H. Nutman T. B. Nutman T B. (2018). Helminth Parasites and Immune Regulation. F1000Res 7 (0), 1685. 10.12688/f1000research.15596.1 PMC6206608 30416709

[B49] Gentilini M. V. Nuñez G. G. Roux M. E. Venturiello S. M. (2011). Trichinella Spiralis Infection Rapidly Induces Lung Inflammatory ResponseThe Lung as the Site of Helminthocytotoxic Activity. Immunobiology 216 (9), 1054–1063. 10.1016/j.imbio.2011.02.002 21411179

[B50] Ghanem K. Abdel-Salam A. Magharby A. (2005). Immunoprophylactic Effect of Probiotic Yoghurt Feeding on Schistosoma Mansoni-Infected Mice. Polish J. Food Nutr. Sci. 14 (2), 123–126.

[B51] Gurram R. K. Zhu J. (2019). Orchestration between ILC2s and Th2 Cells in Shaping Type 2 Immune Responses. Cell Mol Immunol 16 (3), 225–235. 10.1038/s41423-019-0210-8 30792500 PMC6460501

[B52] Hammad H. Lambrecht B. N. (2015). Barrier Epithelial Cells and the Control of Type 2 Immunity. Immunity 43 (1), 29–40. 10.1016/j.immuni.2015.07.007 26200011

[B53] Heath D. D. Lawrence. S. B. (1996). Antigenic Polypeptides of Echinococcus Granulosus Oncospheres and Definition of Protective Molecules. Parasite Immunol. 18 (7), 347–357. 10.1046/j.1365-3024.1996.d01-114.x 9229388

[B54] Hernández-González J. C. Martínez-Tapia A. Lazcano-Hernández G. García-Pérez B. E. Castrejón-Jiménez N. S. (2021). Bacteriocins from Lactic Acid Bacteria. A Powerful Alternative as Antimicrobials, Probiotics, and Immunomodulators in Veterinary Medicine. Animals 11 (4), 979. 10.3390/ani11040979 33915717 PMC8067144

[B55] Herz U. Lacy P. Renz H. Erb K. (2000). The Influence of Infections on the Development and Severity of Allergic Disorders. Curr. Opin. Immunol. 12 (6), 632–640. 10.1016/S0952-7915(00)00155-2 11102765

[B56] Hill C. Guarner F. Reid G. Gibson G. R. Merenstein D. J. Pot B. (2014). Bruno Pot, Lorenzo Morelli, et al.The International Scientific Association for Probiotics and Prebiotics Consensus Statement on the Scope and Appropriate Use of the Term Probiotic. Nat. Rev. Gastroenterol. Hepatol. 11 (8), 506–514. 10.1038/nrgastro.2014.66 24912386

[B57] Hotez P. J. Bethony J. M. Oliveira S. C. Brindley P. J. Loukas A. (2008). Multivalent Anthelminthic Vaccine to Prevent Hookworm and Schistosomiasis. Expert Rev. Vaccin. 7 (6), 745–752. 10.1586/14760584.7.6.745 18665774

[B58] Hurst S. D. Seymour B. W. P. Muchamuel T. Kurup V. P. Coffman R. L. (2001). Modulation of Inhaled Antigen-Induced IgE Tolerance by Ongoing Th2 Responses in the Lung. J. Immunol. 166 (8), 4922–4930. 10.4049/jimmunol.166.8.4922 11290770

[B59] Iraporda C. Errea A. Romanin D. E. Cayet D. Pereyra E. Pignataro O. (2015). Lactate and Short Chain Fatty Acids Produced by Microbial Fermentation Downregulate Proinflammatory Responses in Intestinal Epithelial Cells and Myeloid Cells. Immunobiology 220 (10), 1161–1169. 10.1016/j.imbio.2015.06.004 26101138

[B60] Iraporda C. Romanin D. E. Bengoa A. A. Errea A. J. Cayet D. Foligné B. (2016). Local Treatment with Lactate Prevents Intestinal Inflammation in the TNBS-Induced Colitis Model. Front. Immunol. 7 (DEC), 1–9. 10.3389/fimmu.2016.00651 28082985 PMC5187354

[B61] Islam S. U. (2016). Clinical Uses of Probiotics. Medicine 95 (5), e2658. 10.1097/MD.0000000000002658 26844491 PMC4748908

[B62] Jang S. Lakshman S. Beshah E. Xie Y. Molokin A. Vinyard B. (2017). Flavanol-Rich Cocoa Powder Interacts with Lactobacillus Rhamnossus LGG to Alter the Antibody Response to Infection with the Parasitic Nematode Ascaris Suum. Nutrients 9 (10), 1113. 10.3390/nu9101113 PMC5691729 29023393

[B63] Kazura J. W. Grove D. I. (1978). Stage-specific Antibody-dependent Eosinophil-Mediated Destruction of *Trichinella spiralis* . Nature 274, 588–589. 10.1038/274588a0 672989

[B64] Klementowicz J. E. Travis M. A. Grencis R. K. (2012). Trichuris Muris: A Model of Gastrointestinal Parasite Infection. Semin. Immunopathol 34 (6), 815–828. 10.1007/s00281-012-0348-2 23053395 PMC3496546

[B65] Klose C. S. N. Artis. D. (2016). Innate Lymphoid Cells as Regulators of Immunity, Inflammation and Tissue Homeostasis. Nat. Immunol. 17 (7), 765–774. 10.1038/ni.3489 27328006

[B66] Kouchkovsky D. A. de. Ghosh S. Rothlin C. V. (2017). Negative Regulation of Type 2 Immunity. Trends Immunol. 38 (3), 154–167. 10.1016/j.it.2016.12.002 28082101 PMC5510550

[B67] Kubo M. (2018). Mast Cells and Basophils in Allergic Inflammation. Curr. Opin. Immunol. 54 (October), 74–79. 10.1016/j.coi.2018.06.006 29960953

[B68] Lichtenstein L. Avni-Biron I. Ben-Bassat O. (2016). Probiotics and Prebiotics in Crohn's Disease Therapies. Best Pract. Res. Clin. Gastroenterol. 30 (1), 81–88. 10.1016/j.bpg.2016.02.002 27048899

[B69] Lightowlers M. W. (2010). Fact or Hypothesis: Concomitant Immunity in Taeniid Cestode Infections. Parasite Immunol. 32 (8), no. 10.1111/j.1365-3024.2010.01227.x PMC2913106 20626813

[B130] Lin J. Zhang Y. He C. Dai J. (2018). Probiotics Supplementation in Children With Asthma: A Systematic Review and Meta-Analysis. J Paediatr Child Health 54 (9), 953–961. 10.1111/jpc.14126 30051941

[B70] Loukas A. Hotez P. J. Diemert D. Yazdanbakhsh M. McCarthy J. S. Correa-Oliveira R. (2016). Hookworm Infection. Nat. Rev. Dis. Primers 2. 10.1038/nrdp.2016.88 27929101

[B71] Mackowiak P. A. (2013). Recycling Metchnikoff: Probiotics, the Intestinal Microbiome and the Quest for Long Life. Front. Public Health 1 (NOV), 1–3. 10.3389/fpubh.2013.00052 24350221 PMC3859987

[B72] Maizels R. M. Hewitson J. P. Murray J. Harcus Y. M. Dayer B. Filbey K. J. (2012). Immune Modulation and Modulators in Heligmosomoides Polygyrus Infection. Exp. Parasitol. 132 (1), 76–89. 10.1016/j.exppara.2011.08.011 21875581 PMC6485391

[B73] Maizels R. M. McSorley H. J. Smyth D. J. (2014). Helminths in the Hygiene Hypothesis: Sooner or Later? Clin. Exp. Immunol. 177 (1), 38–46. 10.1111/cei.12353 24749722 PMC4089153

[B74] Maizels R. M. Yazdanbakhsh. M. (2003). Immune Regulation by Helminth Parasites: Cellular and Molecular Mechanisms. Nat. Rev. Immunol. 3 (9), 733–744. 10.1038/nri1183 12949497

[B75] Martínez-Gómez F. Santiago-Rosales R. Ramón Bautista-Garfias C. (2009). Effect of Lactobacillus Casei Shirota Strain Intraperitoneal Administration in CD1 Mice on the Establishment of Trichinella Spiralis Adult Worms and on IgA Anti-T. Spiralis Production. Vet. Parasitol. 162 (1–2), 171–175. 10.1016/j.vetpar.2009.02.010 19269100

[B76] McClemens J. Kim J. J. Wang H. Mao Y.-K. Collins M. Kunze W. (2013). Lactobacillus Rhamnosus Ingestion Promotes Innate Host Defense in an Enteric Parasitic Infection. Clin. Vaccin. Immunol 20 (6), 818–826. 10.1128/CVI.00047-13 PMC3675974 23536695

[B77] McFarland L. V. (2014). Use of Probiotics to Correct Dysbiosis of Normal Microbiota Following Disease or Disruptive Events: A Systematic Review. BMJ Open 4 (8), e005047. 10.1136/bmjopen-2014-005047 PMC4156804 25157183

[B78] McSorley H. J. Maizels R. M. (2012). Helminth Infections and Host Immune Regulation. Clin. Microbiol. Rev. 25 (4), 585–608. 10.1128/CMR.05040-11 23034321 PMC3485755

[B132] Meneghin F. Valentina F. Mameli C. Zuccotti G. V. (2012). Probiotics and Atopic Dermatitis in Children. Pharmaceuticals 5 (7), 727–744. 10.3390/ph5070727 24281709 PMC3763666

[B79] Metchnikoff E. (1908). “The Prolongation of Life Optimistic Studies,” in Ruce Carries. Editors Olshansky S. J. Achenbaum A. Brody J James B.. 2004th ed (London: Springer US).

[B80] Mohamed A. H. Osman G. Y. El-Esawy H. M. I. (2016). Effect of Lactobacillus Sporogenes (Probiotic) on Certain Parasitological and Molecular Aspects in Schistosoma Mansoni Infected Mice. J. Parasit Dis. 40 (3), 823–832. 10.1007/s12639-014-0586-4 27605791 PMC4996199

[B82] Montero R. Ostrosky P. (1997). Genotoxic Activity of Praziquantel1To Dora Valencia (In Memoriam).1. Mutat. Research/Reviews Mutat. Res. 387 (3), 123–139. 10.1016/S1383-5742(97)00027-6 9439709

[B83] Navarro S. Pickering D. A. Ferreira I. B. Jones L. Ryan S. Troy S. (2016). Hookworm Recombinant Protein Promotes Regulatory T Cell Responses that Suppress Experimental Asthma. Sci. Translational Med. 8 (362), 362ra143. 10.1126/scitranslmed.aaf8807 27797959

[B84] Negrão-corrêa D. (2001). Importance of Immunoglobulin E (IgE) in the Protective Mechanism against Gastrointestinal Nematode Infection: Looking at the Intestinal Mucosae. Rev. Inst. Med. Trop. S. Paulo 43 (5), 291–299. 10.1590/S0036-46652001000500011 11696854

[B85] Nithikathkul C. Wongsawad C. (2008). Prevalence of Haplorchis Taichui and Haplorchoides Sp. Metacercariae in Freshwater Fish from Water Reservoirs, Chiang Mai, Thailand. Korean J. Parasitol. 46 (2), 109–112. 10.3347/kjp.2008.46.2.109 18552549 PMC2532605

[B86] Oliveira-Sequeira T. C. G. David É. B. Ribeiro C. Guimarães S. Masseno A. P. B. Katagiri S. (2014). EFFECT of Bifidobacterium Animalis on Mice Infected with Strongyloides Venezuelensis. Rev. Inst. Med. Trop. S. Paulo 56 (2), 105–109. 10.1590/S0036-46652014000200003 24626410 PMC4085849

[B87] Orel R. Trop T. K. (2014). Intestinal Microbiota, Probiotics and Prebiotics in Inflammatory Bowel Disease. World. J. Gasteroentrol. 20 (33), 11505–11524. 10.3748/wjg.v20.i33.11505 PMC4155344 25206258

[B88] Pais P. Almeida V. Yılmaz M. Teixeira M. C. (2020). Saccharomyces Boulardii: What Makes it Tick as Successful Probiotic? J. Fungi 6 (2), 78–15. 10.3390/jof6020078 PMC7344949 32512834

[B89] Pearce E. J. MacDonald. A. S. (2002). The Immunobiology of Schistosomiasis. Nat. Rev. Immunol. 2 (7), 499–511. 10.1038/nri843 12094224

[B90] Pivetti-Pezzi P. (2009). Ocular Toxocariasis. Int. J. Med. Sci. 2 (3), 129–130. 10.7150/ijms.6.129 PMC2659485 19319231

[B91] Plaza-Diaz J. Ruiz-Ojeda F. J. Gil-Campos M. Gil A. (2019). Mechanisms of Action of Probiotics. Adv. Nutr. 10, S49–S66. 10.1093/advances/nmy063 30721959 PMC6363529

[B92] Prommi A. Prombutara P. Watthanakulpanich D. Adisakwattana P. Kusolsuk T. Yoonuan T. (2020). Intestinal Parasites in Rural Communities in Nan Province, Thailand: Changes in Bacterial Gut Microbiota Associated with Minute Intestinal Fluke Infection. Parasitology 147 (9), 972–984. 10.1017/S0031182020000736 32364103 PMC10317732

[B93] Rausch S. Huehn J. Kirchhoff D. Rzepecka J. Schnoeller C. Pillai S. (2008). Functional Analysis of Effector and Regulatory T Cells in a Parasitic Nematode Infection. Infect. Immun. 76 (5), 1908–1919. 10.1128/IAI.01233-07 18316386 PMC2346705

[B94] Reynolds L. A. Finlay B. B. Maizels R. M. (2015). Cohabitation in the Intestine: Interactions Among Helminth Parasites, Bacterial Microbiota, and Host Immunity. J.I. 195 (9), 4059–4066. 10.4049/jimmunol.1501432 PMC4617609 26477048

[B95] Reynolds L. A. Smith K. A. Filbey K. J. Harcus Y. Hewitson J. P. Redpath S. A. (2014). Commensal-Pathogen Interactions in the Intestinal Tract. Gut Microbes 5 (4), 522–532. 10.4161/gmic.32155 25144609 PMC4822684

[B96] Rogan M. T. Craig P. S. Zehyle E. Masinde G. Wen H. Zhou P. (1992). *In Vitro* Killing of Taeniid Oncospheres, Mediated by Human Sera from Hydatid Endemic Areas. Acta Tropica 51 (3–4), 291–296. 10.1016/0001-706X(92)90047-2 1359755

[B97] Ruiz-Manzano R. A. Hernández-Cervantes R. Río-Araiza V. H. D. Palacios-Arreola. M. I. Elizabeth Nava-Castro K. Morales-Montor J. (2019). Immune Response to Chronic Toxocara Canis Infection in a Mice Model. Parasite Immunol. 41 (12), 1–11. 10.1111/pim.12672, 31557337

[B133] Rusu E. Enache G. Cursaru R. Alexescu A. Radu R. Onila O. (2019). Prebiotics and Probiotics in Atopic Dermatitis (Review). Exp. Ther. Med. 18 (2), 926–931. 10.3892/etm.2019.7678 31384325 PMC6639913

[B98] Ryan S. M. Eichenberger R. M. Ruscher R. Giacomin P. R. Loukas A. (2020). Harnessing Helminth-Driven Immunoregulation in the Search for Novel Therapeutic Modalities. Plos Pathog. 16 (5), e1008508–20. 10.1371/journal.ppat.1008508 32407385 PMC7224462

[B99] Sahputra R. Ruckerl D. Couper K. N. Muller W. Else K. J. (2019). The Essential Role Played by B Cells in Supporting Protective Immunity against Trichuris Muris Infection Is by Controlling the Th1/Th2 Balance in the Mesenteric Lymph Nodes and Depends on Host Genetic Background. Front. Immunol. 10 (December), 1–14. 10.3389/fimmu.2019.02842 31921120 PMC6915098

[B100] Sánchez B. Delgado S. Blanco-Míguez A. Lourenço A. Gueimonde M. Margolles A. (2017). Probiotics, Gut Microbiota, and Their Influence on Host Health and Disease. Mol. Nutr. Food Res. 61 (1), 1600240–1600315. 10.1002/mnfr.201600240 27500859

[B101] Santos J. d. F. M. Vasconcelos J. Souza J. R. d. Coutinho E. d. M. Montenegro S. M. L. Azevedo-Ximenes E. (2004). The Effect of Zymomonas Mobilis Culture on Experimental Schistosoma Mansoni Infection. Rev. Soc. Bras. Med. Trop. 37 (6), 502–504. 10.1590/s0037-86822004000600015 15765603

[B102] Saracino M. P. Vila C. C. Cohen M. Gentilini M. V. Falduto G. H. Calcagno M. A. (2020). Cellular and Molecular Changes and Immune Response in the Intestinal Mucosa during Trichinella Spiralis Early Infection in Rats. Parasites Vectors 13 (1), 1–19. 10.1186/s13071-020-04377-8 33023672 PMC7539519

[B103] Setiawan T. Metwali A. Blum A. M. Ince M. N. Urban J. F. Elliott D. E. (2007). Heligmosomoides Polygyrus Promotes Regulatory T-Cell Cytokine Production in the Murine Normal Distal Intestine. Infect. Immun. 75 (9), 4655–4663. 10.1128/IAI.00358-07 17606601 PMC1951154

[B104] Shapouri-Moghaddam A. Mohammadian S. Vazini H. Taghadosi M. Esmaeili S. A. Mardani F. (2018). Macrophage Plasticity, Polarization, and Function in Health and Disease. J. Cel Physiol 233, 6425–6440. 10.1002/jcp.26429 PMC13482560 29319160

[B105] Shubber E. K. Salih H. (1987). CYTOGENETIC STUDIES ON BLOOD LYMPHOCYTES FROM PATIENTS WITH SCHISTOSOMA MANSONI. Jpn. J. Med. Sci. Biol. 40 (4), 137–145. 10.7883/yoken1952.40.137 2455084

[B129] Sivan A. Corrales L. Hubert N. Williams J. B. Aquino-Michaels K. Earley Z. M. (2015). Commensal Bifidobacterium Promotes Antitumor Immunity and Facilitates Anti-PD-L1 Efficacy. Science 350 (6264), 1084–1089. 26541606 10.1126/science.aac4255 PMC4873287

[B106] Solano-Aguilar G. Shea-DonohueShea-Donohue T. Madden K. B. Quinoñes A. Beshah E. Lakshman S. (2018). Bifidobacterium Animalis Subspecies Lactis Modulates the Local Immune Response and Glucose Uptake in the Small Intestine of Juvenile Pigs Infected with the Parasitic Nematode Ascaris Suum. Gut Microbes 9 (5), 1–15. 10.1080/19490976.2018.1460014 30024817 PMC6219643

[B107] Stiemsma L. Reynolds L. Turvey S. Finlay B. (2015). The Hygiene Hypothesis: Current Perspectives and Future Therapies. Immuno. Target. Ther 143, 143. 10.2147/itt.s61528 PMC4918254 27471720

[B108] Svetić A. Madden K. B. Zhou X. D. Lu P. Katona I. M. Finkelman F. D. (1993). A Primary Intestinal Helminthic Infection Rapidly Induces a Gut-Associated Elevation of Th2-Associated Cytokines and IL-3. J. Immunol. (Baltimore, Md 150 (8 Pt 1), 3434–3441. 8468481

[B109] Tan J. McKenzie C. Potamitis M. Thorburn A. N. Mackay C. R. Macia L. (2014). The Role of Short-Chain Fatty Acids in Health and Disease. Adv. Immunol. 121, 91–119. 10.1016/B978-0-12-800100-4.00003-9 24388214

[B110] Tantisira K. G. Silverman E. S. Mariani T. J. Xu J. Richter B. G. Klanderman B. J. (2007). FCER2: A Pharmacogenetic Basis for Severe Exacerbations in Children with Asthma. J. Allergy Clin. Immunol. 120 (6), 1285–1291. 10.1016/j.jaci.2007.09.005, 17980418

[B111] Taylor M. H. O'Connor P. Keane C. T. Mulvihill E. Holland C. (1988). The Expanded Spectrum of Toxocaral Disease. The Lancet 331 (8587), 692–695. 10.1016/S0140-6736(88)91486-9 2895221

[B112] Temsahy M. M. E. Ibrahim I. R. Mossallam S. F. Mahrous H. Bary A. A. Salam S. A. A. (2015). Evaluation of Newly Isolated Probiotics in the Protection against Experimental Intestinal Trichinellosis. Vet. Parasitol. 214 (3–4), 303–314. 10.1016/j.vetpar.2015.08.029 26386829

[B113] Torben W. Ahmad G. Zhang W. Nash S. Le L. Karmakar S. (2012). Role of Antibody Dependent Cell Mediated Cytotoxicity (ADCC) in Sm-P80-Mediated Protection against Schistosoma Mansoni. Vaccine 30 (48), 6753–6758. 10.1016/j.vaccine.2012.09.026 23000221 PMC3488153

[B114] Urban J. F. Katona I. M. Finkelman F. D. (1991). Heligmosomoides Polygyrus: CD4+ but Not CD8+ T Cells Regulate the IgE Response and Protective Immunity in Mice. Exp. Parasitol. 73 (4), 500–511. 10.1016/0014-4894(91)90074-7 1683629

[B115] Urban J. F. Nielsen M. K. Gazzola D. Xie Y. Beshah E. Hu Y. (2021). An Inactivated Bacterium (Paraprobiotic) Expressing Bacillus Thuringiensis Cry5B as a Therapeutic for Ascaris and Parascaris Spp. Infections in Large Animals. One Health 12, 100241. 10.1016/j.onehlt.2021.100241 33889707 PMC8048022

[B116] Venturiello S. M. Giambartolomei G. H. Costantino S. N. (1993). Immune Killing of newbornTrichinellalarvae by Human Leucocytes. Parasite Immunol. 15 (10), 559–564. 10.1111/pim.1993.15.10.559 7877832

[B117] Vogt C. M. Armúa-Fernández M. T. Tobler K. Hilbe M. Aguilar C. Ackermann M. (2018). Oral Application of Recombinant Bacillus Subtilis Spores to Dogs Results in a Humoral Response against Specific Echinococcus Granulosus Paramyosin and Tropomyosin Antigens. Infect. Immun. 86 (3), 1–16. 10.1128/IAI.00495-17 PMC5820962 29229735

[B118] Walcher D. L. Cruz L. A. X. de Lima Telmo P. Martins L. H. R. da Costa de Avila L. F. Berne M. E. A. (2018). Lactobacillus Rhamnosus Reduces Parasite Load on Toxocara Canis Experimental Infection in Mice, but Has No Effect on the Parasite *In Vitro* . Parasitol. Res. 117 (2), 597–602. 10.1007/s00436-017-5712-7 29243027

[B119] Wammes L. J. Mpairwe H. Elliott A. M. Yazdanbakhsh M. (2014). Helminth Therapy or Elimination: Epidemiological, Immunological, and Clinical Considerations. Lancet Infect. Dis. 14 (11), 1150–1162. 10.1016/S1473-3099(14)70771-6 24981042

[B120] Wang C. H. Bell R. G. (1987). *Trichinella spiralis*: Intestinal Expression of Systemic Stage-specific Immunity to Newborn Larvae. Parasite Immunol. 9 (4), 465–475. 10.1111/j.1365-3024.1987.tb00523.x 3627826

[B121] Wang D. Gong Q.-L. Huang H.-B. Yang W.-T. Shi C.-W. Jiang Y.-L. (2020). Protection against Trichinella Spiralis in BALB/c Mice via Oral Administration of Recombinant Lactobacillus Plantarum Expressing Murine Interleukin-4. Vet. Parasitol. 280 (January), 109068. 10.1016/j.vetpar.2020.109068 32278937

[B131] Wei X. Jiang P. Liu J. Sun S. Zhu L. (2020). Association Between Probiotic Supplementation and Asthma Incidence in Infants: A Meta-Analysis of Randomized Controlled Trials. J. Asthma 57 (2), 167–178. 10.1080/02770903.2018.1561893 30656984

[B122] Weill F. S. Cela E. M. Paz M. L. Ferrari A. Leoni J. Maglio D. H. G. (2013). Lipoteichoic Acid from Lactobacillus Rhamnosus GG as an Oral Photoprotective Agent against UV-Induced Carcinogenesis. Br. J. Nutr. 109 (3), 457–466. 10.1017/S0007114512001225 22874095

[B123] Wiedemann M. Voehringer D. (2020). Immunomodulation and Immune Escape Strategies of Gastrointestinal Helminths and Schistosomes. Front. Immunol. 11 (September), 1–13. 10.3389/fimmu.2020.572865 33042153 PMC7527441

[B124] Wilkes C. P. Bleay C. Paterson S. Viney M. E. (2007). The Immune Response during a Strongyloides Ratti Infection of Rats. Parasite Immunol. 29 (7), 339–346. 10.1111/j.1365-3024.2007.00945.x 17576363 PMC2042580

[B125] Wu Z. Wang L. Tang Y. Sun X. (2017). Parasite-Derived Proteins for the Treatment of Allergies and Autoimmune Diseases. Front. Microbiol. 8 (NOV), 1–13. 10.3389/fmicb.2017.02164 29163443 PMC5682104

[B126] Yazdanbakhsh M. Kremsner P. G. Van Ree R. (2002). Allergy, Parasites, and the Hygiene Hypothesis. Science 296 (5567), 490–494. 10.1126/science.296.5567.490 11964470

[B127] Yousif S. Y. Ali A. A. (2020). Effect of Probiotic Acidophilus Plus against Infection with Secondary Hydatid Disease in BALB/c Mice. Ijvs 34 (1), 115–121. 10.33899/IJVS.2019.125613.1104

[B128] Zheng J. Wittouck S. Salvetti E. Franz C. M. A. P. Harris H. M. B. Mattarelli P. (2020). A Taxonomic Note on the Genus Lactobacillus: Description of 23 Novel Genera, Emended Description of the Genus Lactobacillus Beijerinck 1901, and Union of Lactobacillaceae and Leuconostocaceae. Int. J. Syst. Evol. Microbiol. 70 (4), 2782–2858. 10.1099/ijsem.0.004107 32293557

[B81] Zowail M. E. M. Osman G. Y. Mohamed A. H. El-Esawy H. Y. (2012). Protective role of *Lactobacillus sporogenes* (probiotic) on chromosomal aberrations and DNA fragmentation in *Schistosoma mansoni* infected mice. J. Exp. Biol. (Zoo.) 8 (1), 121–130.

